# The fight against polio through the NO-DO newsreels during the Francoism period in Spain

**DOI:** 10.1371/journal.pone.0225324

**Published:** 2019-11-21

**Authors:** José Tuells, Berta Echániz-Martínez

**Affiliations:** Cátedra Balmis de Vacunología. University of Alicante, Alicante (Spain); University of California San Francisco, UNITED STATES

## Abstract

The weekly NO-DO newsreels, official and of obligatory projection in cinemas, held an information monopoly during the Francoist dictatorship (1943-1975) in Spain. The NO-DO was used as an instrument of indoctrination and legitimation, building a discourse based on the regime’s needs and interests. In this study, we examined newsreels on medical subjects related to vaccine-preventable diseases. A majority of reports centred on poliomyelitis, and two differentiated periods could be defined, coinciding with the evolution of the Franco regime’s foreign policy. The first period reflected the regime’s era of isolation and referred to polio as a foreign disease, with the NO-DO showing the US initiatives to fight against it, as it had become the scientific model to follow. Subsequently, the ambiguities of the news related to the disease reflected the dictatorship’s refusal to confront the epidemic suffered by the Spanish population until the vaccination campaigns began in 1963. Even then, the consequences that the negligent management of the disease had for many families were concealed. Meanwhile, the image of a modernized country concerned about national public health was legitimized.

## Introduction

In September 1942, an institution called "News and Documentaries" *(Noticiarios y Documentales*) was established in Spain, and soon became popularly known as NO-DO. The following year, under the slogan "The whole world within reach of the Spanish (people)", it began to broadcast weekly news, quickly becoming the propaganda media par excellence of the Franco regime [1].

From that moment on, the newsreels produced by the NO-DO were the only ones the Spanish population had access to, and their showing was mandatory in all movie theatres. The regime had thus set up an instrument to control and centralize audiovisual information, and it applied strict methods of disinformation and content censorship, as had been the case in other totalitarian countries [2–3].

Foreign newsreels that had been regularly shown until then, such as those from Fox Movietone (USA), UFA News (Germany) or Luce (Italy), were eliminated and replaced with the newly minted, nationally-produced NO-DO newsreels, directed towards serving the political, economic and social interests of the dictatorship. Furthermore, NO-DO became the only institution that was authorized to exchange filmed news with other countries [4].

For technical reasons, the newsreels did not last for more than 10 minutes and were divided into "sections". The news did not use explicit chronological references and this sometimes led to intentional time lags between the moment an event actually occurring and the moment it was shown on the news [5].

More than three decades after, in January 1976, the compulsory nature of NODO broadcasting was abolished, although it remained active until May 1981, becoming part of the archives of the Spanish Film Library [6].

The beginnings of the NO-DO newsreels coincided with devastating socioeconomic realities linked to the autarky of initial Francoism. The regime’s interventionist policy extended to all fields, giving priority to a rigorous system of wide-ranging rationing and restriction [7]. This course of action was inefficient (economic stagnation, subsistence crisis and black market trade), and resulted in stark inequalities, in which the majority of the population experienced hunger, lived in overcrowded conditions and had no access to basic products or medicine, with illiteracy rates reaching 35% [8]. These conditions of shortage and scarcity were immediately reflected in rising infant mortality and mortality due to infectious diseases, with epidemic outbreaks of smallpox, diphtheria or exanthematic typhus [9]. The fresh NO-DO newsreels hid those social realities. They were clearly designed to indoctrinate and focused on constructing stories tailored to the needs of the newly constituted order, offering straightforward and unquestionable models of representation. NO-DO newsreels attempted to convey positive images of Franco’s regime to foster popular approval and to exalt present times that were recovering a glorious past [10].

In recent years, several publications have paid special attention to NO-DO newsreels on topics of scientific diffusion and public health, thus broadening the scope of research in this field [11–16]. The present work follows that line of research and, based on a historical-critical method, it analyzes the news related to immunopreventable diseases in order to study the political evolution of the regime, using as a paradigmatic example: those news dealing with polio. Through them, we will see how the projected image of the preventive fight against this disease, both in the USA and in Spain, was used by Francoism machinery to build a new national identity, also in the international context, as that turned its foreign policy and the idea of a modern state concerned with the welfare of its population was forged, evoked a resurgence of the country, although the social reality was a very different one [17].

## Materials and methods

The NO-DO’s news related to science and technological development, including some news from abroad, constitute an exceptional source for learning about how these issues were perceived through the self-serving image they offered. Newsreels thus became a mechanism for scientific literacy in the hands of the state [18]. This was not in vain, as during those years, the dissemination of health information became a priority, and for good reasons. Slots were dedicated to various topics such as breastfeeding, prenatal hygiene, nutrition, tuberculosis and vaccines, among others. Thus, the long period in which NO-DO was present in society’s collective imagination coincided with the implementation of vaccination campaigns that were used by the regime to convey an image of modernity and progress.

Considering the chronological span covered by NO-DO newsreels allows illustrating how the information offered to Spanish society evolved with political events, and how the NO-DO was used as a state instrument to legitimize, manipulate and create symbolic references for several different generations.

The search on the Webpage of the NO-DO archive (http://www.rtve.es/alacarta/videos-audios/noticiarios-nodo/) was performed by delimiting a specific chronological period: the Francoist dictatorship, choosing as start and end dates: 1943 (year of first screening) and 1975 (year of Franco's death). Once the chronological framework was selected, a search was made based on the following keywords: vacuna, vacunación, campaña, cólera, difteria, paperas, gripe, polio, poliomielitis, rubéola, sarampión, tétanos, viruela, enfermedad, laboratorio, parálisis infantil, rehabilitación, salud, salud pública, virus, Seguro Obligatorio de Enfermedad, Dirección General de Sanidad. However, after this first search, it was decided to apply an exclusion criterion: eliminate those results that did not directly address the subject of study. While adding other words that the analysis of the news itself had thrown: salud, enfermedad, San Rafael, Niño Jesús. Finally, those NO-DO newsreels that appeared duplicates were discarded, resulting from two or more keywords.

Specifically, among all the newsreels that referred to immuno-preventable diseases, special attention was paid to those dealing with poliomyelitis. Studying these reports allowed for, among other things, the evaluation of how certain Western countries, especially the United States, were presented as models of scientific power in agreement with the political alignment of the dictatorship after the end of Second World War, as the Spanish regime tried to normalize its international relations with a new image. Lastly, this study offered the opportunity to delve into the information treatment given to poliomyelitis, inside and outside of the country, as we review the consequences of the regime’s management of this disease on Spanish society.

## Results and discussion

After applying the designed search strategies, the search of the online NO-DO archive with the keywords mentioned above resulted in a total of 51 videos that referred to vaccine-preventable diseases (Table 1), with most of them (82.3%) produced in the 1950s and 1960s. Most of the news analyzed were of Spanish origin (70.5%), while the rest came from other countries, mostly from the United States (13.7%).

**Table 1:** Number, date of issue, origin and subject(s) of the newsreels analyzed in NO-DO.

Nº	VIDEO	DATE	ORIGIN	SUBJECT(S)
1	6	08/02/1943	Germany	Typhus
2	35A	30/08/1943	Germany	Malaria
3	177B	27/05/1946	Spain	Tuberculosis
4	255B	24/11/1947	Spain	Tuberculosis / Inauguration
5	269A	01/03/1948	USA	Polio
6	350A	19/09/1949	USA	Polio
7	352A	03/10/1949	Spain	Tuberculosis / Inauguration
8	373B	27/02/1950	Spain	Polio
9	423A	12/02/1951	Spain	Flu
10	477B	25/02/1952	Spain	Rabies / Cholera
11	504A	01/09/1952	Spain	Inauguration / Childhood
12	520A	22/12/1952	Spain	Polio
13	522A	05/01/1953	Spain	Polio
14	548B	06/07/1953	Spain	Tuberculosis / Inauguration
15	555A	24/08/1953	Spain	Tuberculosis / Inauguration
16	630A	31/01/1955	USA	Polio
17	633B	21/02/1955	USA	Polio
18	642A	25/04/1955	USA	Polio
19	648B	06/06/1955	Spain	Polio / Inauguration
20	685B	20/02/1956	Italy	Polio
21	690A	26/03/1956	Canada	Polio
22	696A	07/05/1956	Spain	Polio / Inauguration
23	696B	07/05/1956	Spain	Influenza / polio / encephalitis / yellow fever / smallpox / rabies /
24	730A	31/12/1956	Spain	Polio
25	732A	14/01/1957	Spain	Polio
26	767A	16/09/1957	Spain	Flu
27	783A	06/01/1958	Spain	Polio
28	793B	17/03/1958	USA	Polio
29	822A	06/10/1958	Spain	Polio
30	836A	12/01/1959	Spain	Polio
31	850A	20/04/1959	Spain	Tuberculosis / Inauguration
32	896B	07/03/1960	USA	Polio
33	939C	02/01/1961	Spain	Polio
34	964C	26/06/1961	Spain	Polio
35	975C	11/09/1961	China	Cholera
36	994A	22/01/1962	Germany	Smallpox
37	1036B	12/11/1962	Italy	Smallpox
38	1041B	17/12/1962	France	Flu
39	1049B	11/02/1963	Spain	Polio
40	1094B	23/12/1963	Spain	Polio
41	1099B	27/01/1964	Spain	Polio
42	1145C	14/12/1964	Spain	Polio
43	1158B	15/03/1965	Spain	Diphtheria / tetanus / pertussis / polio
44	1197C	13/12/1965	Spain	Polio / Rehabilitation
45	1202C	17/01/1966	Spain	Polio
46	1226A	04/07/1966	Spain	Tuberculosis
47	1254C	16/01/1967	Spain	Polio
48	1302A	18/12/1967	Spain	Polio
49	1306B	15/01/1968	Spain	Polio
50	1432B	15/06/1970	Spain	Polio
51	1626A	11/03/1974	England	Flu

### Foreign affairs, public health and infectious diseases

After the end of the Second World War, Spain was relegated from all international forums, as expressed in the resolutions of the United Nations General Assembly in 1946. Alberto Martín Artajo, the representative of the Catholic faction in Spain, was elected as Minister of Foreign Affairs (1945-1957) to face this isolation [19,20]

In view of the regime’s pressing need to build a new political profile that would erase any memory of its old friendships with Italian fascism and German Nazism (relations that were reflected in the news of 1943: NO-DO 6 and 35) a model of a country was designed that was in tune with the interests of the allies.

Spain thus became the champion in the defense of Catholicism and classical conservatism and a profound anti-Communist entity. The new international political configuration forced the regime to focus its gaze on Portugal, Ibero-America or the Arab countries [21].

In addition, the grand speeches of praise were now directed towards the USA, as the North American power had become the model of scientific, technological and military progress to be admired in the late 1940s, as observed in the media coverage that focused on extolling the fight against polio in that country (NO-DO 269A, 1948 and NO-DO 350A, 1949), as will be shown in more detail below.

However, the Cold War and the Francoist diplomatic policy contributed to the ending of this period of isolation. The initial reservations of the USA towards the Franco regime were dissipated in favor of a bilateral approach that found Spain as an ally in the fight against Communism and allowed it to be included in the American defensive machinery, with the installation of military bases and logistics [22].

At the same time, the process of incorporation of Spain to different international organizations helped to enhance the image of modernity that the regime wanted to provide, with Spain joining various international organizations, thus enhancing an image of modernity.

In this sense, the 1953 agreements with the US and its subsequent renovations represented a turning point in Spanish foreign policy, which was subordinated to the North American power; these agreements brought economic, military and cultural consequences, through educational and technical exchange programs [23].

Two years prior, Spain had joined the WHO, and in 1956, a WHO congress was held in Madrid, as publicized in the NO-DO. The latest discoveries and laboratory trials were reported, highlighting the scientific collaboration between countries: "flu, poliomyelitis, encephalitis, yellow fever, smallpox or rabies present problems that are studied by Spanish and foreign experts who are locating and identifying pathogenic mechanisms" (NO-DO 696B, 1956).

This new image of Spain on the international stage coincided with the appointment of Fernando María Castiella as Minister of Foreign Affairs (1957-1969). With an adequate profile for the technocratic period that had just began, his diplomatic design included an rapprochement to Europe and a solid loyalty to the Western bloc [24].

This international political configuration can be observed in several news items, which revealed a type of message aimed at warning the population that terrible diseases existed in other countries, such as influenza and cholera, placing the origin of both epidemics in "Communist China" (NO-DO 767A, 1957 and NO-DO 795C, 1961), or even isolated cases of smallpox in Germany (NO-DO 994A, 1962) or Italy (NO-DO 1036B, 1962).

These health alerts concluded, however, with messages of reassurance for the population, in which the European and American authorities were presented as guardians of global health stability, while insistently demonstrating that Spain was up to the scientific standards of the other powers (NO-DO 767A, 1957 and NO-DO 994A, 1962).

For its part, the regime took advantage of the newsreels to present itself as a welfare state, constantly concerned about the health of the population. This discourse can be observed in certain issues, which was clearly descriptive and apologetic, and broadcasts aimed at negating any signs of historical continuity with the previous republican period, noting common elements that were repeated across most of them. In order to enhance a blatantly positive vision of health development and reinforce a certain value system, these broadcasts were chosen to open the news, preceded by a warm reception given to the dictator, "with the uttermost devotion and respect" (NO DO 504A, 1952).

Thus, we emphasize the detailed descriptions were given of investment costs, the large number of available beds, the splendor of occupied spaces, or the modern equipment that had been acquired. Repeated messages relating to "modern facilities" (NO-DO 648B, 1955), "modern elements" (NO-DO 696A, 1956) or "the most modern advances" (NO-DO 504A, 1952), turned the country’s modernity and progress into a subject of permanent and obsessive NO-DO discourse as it strove to justify the regime.

In this work of legitimation, the news on the battle against tuberculosis is worth noting, an endeavor that was used by Franco since the beginning of the civil war, with clearly propagandistic intentions following the creation of the National Anti-tuberculosis Board (Patronato Nacional Antituberculoso) [25]. The incidence of this disease, as other infectious diseases, increased after the civil war due to malnutrition, overcrowding and poverty. The Board, as reflected in the images studied, helped to strengthen the dictatorship’s legitimacy as a guardian of the population’s health and a staunch fighter of diseases, adopting measures of assistance (building or rehabilitating sanatoriums and facilities) and preventive measures (dispensaries and childcare centres) [26]. In several analyzed news the dictator appeared inaugurating anti-tuberculosis sanatoriums in different cities, with a clear speech of glorification of the regime: Albacete (NO-DO 255B, 1947), Orense (NO-DO 352A, 1949), Huesca (NO-DO 548B, 1953), San Sebastián (NO-DO 555A, 1953) or Zaragoza (NO-DO 850A, 1959). The process of legitimizing the dictatorship is evident when newsreels collected images of an idealized child's stay in a coastal city’s preventorium. Several women who spent time at the Guadarrama preventorium during their childhood are currently taking part in a lawsuit against Franco regime crimes in Argentina: they are giving testimonies on inhuman punishments received during their stay. These declarations contrast with the desired image given in the news, "where Spanish girls find health and strength amid their many childhood games" (NO-DO 1226A, 1966).

Vaccination was also included in the second type of measures. The National Plan to Combat Tuberculosis, set up by the General Directorate of Health, was another initiative presented by the NO-DO as part of national scientific success, emphasizing the ideal of a protected childhood. An illustration of this is a newsreel that gave a detailed description of a girl’s trip to Madrid. This girl represented the millionth person to be vaccinated with the BCG vaccine (NO-DO 1226A, 1966).

### Poliomyelitis in the NO-DO newsreels

Most of the news related to vaccine-preventable diseases referred to polio (64.7%). We chose to study the newsreels from a two-sided perspective, depending on their origin and the context in which the fight against polio was narrated. Thus, the discussion is divided into a) the international fight against polio, and b) the fight against polio in Spain.

#### International fight against polio

Poliomyelitis, a highly contagious viral disease caused by poliovirus which affects the central nervous system, emerged as an epidemic disease in the late nineteenth century, first in Europe –in Sweden in 1881- and in the following decade in the United States and Canada. From that moment on, it became a serious public health issue [27–35]. The US developed many types of research to find solutions to the epidemic that was spreading in the country. It was in the U.S. that the first civil associations appeared under state protection as a social response to the disease; the most famous was the National Foundation for Infantile Paralysis (NFIP), created in 1938 and promoted by Franklin D. Roosevelt, which was later became popularly known as the "March of Dimes".

As we have observed, as the regime's foreign policy evolved from a social and scientific perspective, its referents also changed. The NO-DO discourse exalted the North American power in its fight against this illness and the research and scientific advances that were implemented for finding a vaccine that could end this epidemic (NO-DO 350A, 1949)

Thus, the news echoed how the "March of Dimes" day unfolded, including mass parades, donations as well as enthusiastic and optimistic faces (NO-DO 269A, 1948); it also showed how Vice-President Richard Nixon collaborated in a charity campaign to finance scientific research (NO-DO 630A, 1955).

To bolster Spanish collective imagination, the NO-DO also projected fashion shows that were used to raise funds to fight the epidemic or galas attended by very popular artists at the moment. These artists helped to publicize the problem and were shown alongside girls affected by the disease, such as Mary Kosloski (NO-DO 633B, 1955) or the Lindy and Sandy Solomon twins with Marcel Marceau, the mime (NO-DO 793B, 1958). These were motivational messages, full of hope and overcoming, that had nothing to do with the ravages that the disease was causing in Spain.

At the University Of Pittsburgh School of Medicine, Jonas Salk, based on previous research, and supported by the NFIP, began developing a vaccine created with killed (inactivated) viruses. Not without controversy, and after numerous trials, the epidemiologist Thomas Francis Jr. and Jonas Salk finally announced the existence of a new vaccine in a massive press conference (April 12, 1955) [36,37]. The details of the event, from the hand of the "hero of this great conquest for life", alluding to Jonas Salk, would be provided to the Spanish population through the NO-DO. The report included images of the trials conducted by Salk himself with monkeys; the press conference starring both doctors; and the long queues of school children waiting to be vaccinated and receiving their vaccination card for subsequent follow-up (NO-DO 642A, 1955). The success of the Salk vaccine was dimmed by the Cutter incident [38–40] and by the discovery of a new, orally administered attenuated vaccine that had resulted from the collaboration of several researchers: Koprowski, Cox and Sabin were able to produce different oral polio vaccines [41,42]. Even one of the field-tested trials of the oral vaccine carried out in Florida was shown in the news (NO-DO 896B, 1960). Thus, through these hopeful images, thanks to the news about scientific achievements that control the disease, the USA became, for the francoism, in the power to look at, in the country to be imitated.

#### The fight against polio in Spain

In Spain, since the late 1940s, poliomyelitis had been an emerging disease that took on a marked epidemic nature that became more acute, especially since the 1950s, with the highest rates of morbidity and mortality recorded in1958 [43,44]. Precisely that same year the 5th International European Symposium dedicated to polio was celebrated in Madrid, an event that was used by the authorities with a clear propagandistic purpose, as part of the effort by the regime to be accepted in the international context (NO-DO 822A, 1958).

Until that moment, in the newsreels, the references to polio evoked other countries, other distant and foreign struggles. The NO-DO projected the image of a disease that seemed foreign to Spain, although cases of affected people continued to rise in an unstoppable manner. Only a few videos mentioned the inauguration of specific poliomyelitis pavilions or the acquisition of steel lungs, without explaining their medical utility. They appeared rather as one more piece of a showcase to enhance and improve the image of efficacy fostered by the regime (NO-DO 373B, 1950 and NO-DO 648B, 1955).

Spain intended these events to match the scientific or modern levels of its surrounding countries, championing the fight against a disease that seemed proper to developed countries. Franco’s regime, however, refused to admit that polio was a serious epidemic problem of the Spanish population [45].

Also, this persistent denial of social realities coincided with internal disputes that were taking place between the families of the regime to control Preventive Medicine, and these clashes would result in two simultaneous campaigns of mass immunization against polio in 1963, almost a decade after the Salk vaccine was available [46]

Previously, the vaccines had been supplied by the General Directorate of Health to three sectors of the population at different rates: the poor cared for by charity through Social Assistance, low income and wealthy families. Nonetheless, in that year, two different associations competed to brandish the success of their respective vaccination campaign. These were: the Mandatory Sickness Insurance (NO DO 1049B, 1963) -under the control of the Falangists- using the injectable Salk vaccine, and the General Directorate of Health -led by Catholic military doctors- using the oral vaccine Sabin (NO-DO 1094B, 1963 and NO-DO 1158B, 1965) [47]. In this competition (Salk versus Sabin), the oral vaccine won, and from then on, national immunization campaigns were based on the Sabin vaccine.

In all the newsreels that referred to vaccine-preventable diseases, also in the case of polio, the language was utilized and manipulated by the dictatorship to perpetuate its own value system. If we pay close attention to the discourse, we will find that it is always dotted with a vocabulary that seeks to reach most of Spanish society, as a transmitter of ideas that the population could easily identify with [48].

Thus, it is observed how the NO-DO news on polio in Spain was narrated using vague terms, with no true relationship whatsoever with the children affected, or medical clarifications or contributions. In fact, in many of the analyzed broadcasts, the name of the disease was not mentioned, it is not heard, it is omitted. Instead, the narrator mentions the "sick little people" or "handicapped children", in this way, the regime, by not naming it, silences the epidemic.

When the Francoist government joined the fight against polio, as had previously occurred with other diseases, this was conceived to sustain the image of progress and modernity that would be able to equalize Spain with other modern countries. This is a cruel portrait that collided with the aftermath of thousands of affected families and the disastrous management of the treatment of the disease by the Francoist regime, always avoiding any type of responsibility in this regard. Today, many people affected by the post-polio syndrome, aware of their common demands and claiming social justice have sought a specific associative response based on these shared experiences, offering testimonies of medical-sanitary realities silenced and forgotten by the regime [49].

## Conclusions

Following a historical-critical analysis of the newsreels, the object of this study, we were able to observe how the NO-DO was used, from the day of its creation, as a political element for the legitimization of the Franco dictatorship and as a propaganda instrument that served the interests of the new regime. The information and disinformation in these newsreels evolved at the same pace as the Francoism foreign policy, through a new image of the country based on Catholicism and anti-Communism. In this sense, the study of the news referring to vaccine-preventable diseases, especially those related to the fight against polio, identifies two distinct stages. Thus, after the break with a first stage characterized by international isolation, the Cold War and the diplomatic machinery of the regime contributed to a bilateral rapprochement with the Western countries, especially the United States. A very clear turning point was found in the 1950s, with the integration of Spain into international organizations and agreements signed with the North America power, which had become the scientific and technological model to imitate and extol, as evidenced by the news coming from there. Thus, in this second stage, the NO-DO used this type of news to build a new image of the regime based on the ideas of modernity, prosperity and progress. Through a univocal ideological discourse and with a paternalistic message, the fight against polio was used to defend the idea of Welfare State that would equate Spain with other Western countries. The news about this disease in the Spanish context was imprecise and blurred, as a clear indication of the dictatorship's refusal to face the epidemic problem suffered by the Spanish population. In spite of the first mass vaccination campaigns that began in 1963, the dictatorship’s ultimate aim was to project the image of a country at the forefront of the fight against the disease, silencing and hiding the consequences of its negligent management of the polio epidemic and the drama that it caused for many families.

## References

[1] Rodríguez Tranche R, Sánchez-Biosca V. NO-DO. El tiempo y la memoria. Madrid: Cátedra/Filmoteca Española; 2001. Ellwood S. Spanish newsreels 1943-1973: the image of the Franco regime. Hist. J. Film Radio Telev. 1987;7(3): 225–238.

[2] Rueda Lafford JC, Coronado Ruiz C. La codificación televisiva del franquismo: de la historia del entretenimiento a la historia como entretenimiento. Hist. Crit. 2010;40(1): 170–195.

[3] Rodríguez Tranche R. NO-DO: la memoria documental del franquismo In: Catalá JM, Cerdán J, Torreiro C, coordinators. Imagen, memoria y fascinación. Notas sobre el documental en España. Madrid: Ocho y Medio; 2001.

[4] Rodríguez Mateos A. Un franquismo de cine: la imagen política del Régimen en el noticiario NO-DO (1943-1959). Madrid: Ediciones Rialp; 2008.

[5] Garrido F. El frente del este en el NO-DO: desinformación y propaganda, 1943-1945. Filmhistoria.1993;3(1): 321–337.

[6] Díez Puertas E. Historia social del cine en España. Madrid: Fundamentos; 2003.

[7] Montero Díaz M. La publicidad española durante el franquismo (1939-1975). De la autarquía al consumo. Hispania. 2012;72(240): 205–232.

[8] Arco Blanco M A del. Morir de hambre autarquía, escasez y enfermedad en la España del primer franquismo. Pasado y memoria. 2006;5: 241–258.

[9] Jiménez Lucena I, Ruiz Somavilla MJ, Castellanos Guerrero J. Un discurso sanitario para un proyecto político. La educación sanitaria en los medios de comunicación de masas durante el primer franquismo. Asclepio. 2002; 54 (1): 201–218. 17304714

[10] Rodríguez Tranche R, Sánchez-Biosca V. Los años 50 en NO-DO: de la autarquía al desarrollismo In Yraola A, editor. Historia Contemporánea de España y Cine. Madrid: Ed. Universidad Autónoma de Madrid; 1997 p. 115–124.

[11] Menéndez-Navarro A, Medina-Doménech RM. Ausencia y primor: ‘Mujer’, tecnologías médicas e identidad nacional en el discurso visual de NO-DO In: Amador Carretero P, Ruiz Franco R, editors. Representación, construcción e interpretación de la imagen visual de las mujeres. X Coloquio Internacional de la AEIHM: Madrid: Instituto de Cultura y Tecnología ‘Miguel de Unamuno’; 2003 p. 395–403.

[12] Medina-Doménech RM, Menéndez-Navarro A. Cinematic representations of medical technologies in the Spanish official newsreel, 1943-1970. Public Underst. Sci. 2005;14: 393–408. 10.1177/0963662505056692 16402492

[13] Ramírez Martínez F. Ciencia, tecnología y propaganda: el NO-DO, un instrumento de popularización de la ciencia al servicio del Estado (1943-1964). Actes d'història de la ciència i de la técnica. 2008;1(2): 253–261.

[14] Menéndez Navarro A. Una cámara para nuestro amigo el átomo: la representación de las tecnologías médicas nucleares en NO-DO. Quaderns de Cine. 2009;4: 47–56.

[15] Pulpillo Leiva C. Visión y objetivos de la política exterior del primer franquismo en el Noticiero de España (1939-1941) In: Navajas Zubeldia C, Iturriaga Barco D, editors. Actas del V Congreso Internacional de Historia de Nuestro Tiempo. Logroño: Universidad de La Rioja; 2016 p. 215–231.

[16] Tabernero C. The Changing Nature of Modernization Discourses in Documentary Films. Science in Context. 2018; 31(1): 61–83. 10.1017/S0269889718000066 29580313

[17] Sánchez Biosca V. Los lugares de las memorias franquistas en el NO-DO. ArtCultura. 2009;11 (18): 95–108.

[18] Ordóñez Rodríguez J, Ramírez Martínez F. Los públicos de la ciencia española: un estudio del NO-DO In: Romero de Pablos A, Santesmases Navarro MJ, coordinators. Cien años de política científica en España. Bilbao: Fundación BBVA; 2008 p. 257–292.

[19] Fernández Fernández-Cuesta JM. La información al servicio de la política exterior. La creación de la oficina de información diplomática, respuesta del franquismo al aislamiento internacional (1945-1950). Revista internacional de Historia de la Comunicación. 2013;1(1): 132–154.

[20] Tusell Gómez J. Los cuatro ministros de asuntos exteriores de Franco durante la Segunda Guerra Mundial. Espacio, Tiempo y Forma, Serie V, H. Contemporánea. 1994;7: 323–348.

[21] Eiroa San Francisco M. Urdiendo el tejido exterior para el Nuevo Estado: la política internacional del Primer Franquismo. Historia y Comunicación Social. 2001;6: 203–214.

[22] Delgado Gómez-Escalonilla L. Estados Unidos, ¿soporte del franquismo o germen de la democracia? In: Delgado Gómez Escalonilla L, Martín de la Guardia R, Pardo Sanz R, coordinators. La apertura internacional de España. Entre el franquismo y la democracia, 1953-1986. Madrid: Sílex; 2016 p. 263–307.

[23] Delgado L. ¿“El “amigo americano”? España y Estados Unidos durante el franquismo. Studia histórica. 2003;21: 231–276.

[24] Pardo Sanz R. La política exterior del Franquismo: aislamiento y alineación internacional In: Moreno R, Sevillano F, editors. El Franquismo. Visiones y balances. Alicante: Universidad de Alicante; 1999 p. 93–118.

[25] Molero Mesa J. Enfermedad y previsión social en España durante el primer franquismo (1936-1951). El frustrado seguro obligatorio contra la tuberculosis. Dynamis. 1994;14: 199–225. 11624903

[26] Marín Martínez P. El Preventorio Infantil Antituberculoso de Almería (1945-1965). Dynamis. 1990;10: 275–301. 11638269

[27] Paul RJ. A history of poliomyelitis. New Haven, CT: Yale University Press; 1971.

[28] Wilson D. A crippling fear: experiencing polio in the era of FDR. Bull. Hist. Med. 1988;72: 464–495. 10.1353/bhm.1998.0163 9780450

[29] Rogers N. Dirt and disease: Polio before FDR. New Brunswick: Rutgers University Press; 1992.

[30] Gould A. A summer plague: polio and its survivors. New Haven/ London: Yale University Press; 1995.

[31] Black K. In the shadow of Polio: a personal and social history. Reading: Addison-Wesley; 1996.

[32] Sass E. Polio´s legacy. An oral history. Lanhan/ London: University Press of America; 1996.

[33] Shell M. Polio and its aftermath The paralysis of culture. Cambridge: Harvard U.P.; 2005.

[34] Oshinsky DM. Polio. An American story. Oxford-New York: Oxford University Press; 2005.

[35] Smallman-Raynor M, Cliff A, editors. Poliomyelitis: A world geography Emergence to eradication. Oxford: Oxford University Press, 2006.

[36] Dawson L. The Salk Polio Vaccine Trial of 1954: risks, randomization and public involvement in research. Clin Trials. 2004;1(1):122–30. 10.1191/1740774504cn010xx 16281467

[37] Markel H. April 12, 1955-Tommy Francis and the Salk vaccine. N Engl J Med. 2005;352(14): 1408–1410. 10.1056/NEJMp048250 15814876

[38] Nathanson N, Langmuir AD. The Cutter incident. Poliomyelitis following formaldehyde-inactivated poliovirus vaccination in the United States during the Spring of 1955. II. Relationship of poliomyelitis to Cutter vaccine. 1963. Am J Epidemiol. 1995;15;142(2):109-40. 7598112 10.1093/oxfordjournals.aje.a117611

[39] Offit P. The Cutter Incident: How America’s First Polio Vaccine Led to Today’s Growing Vaccine Crisis. New Haven: Yale University Press; 2005.

[40] Day A. 'An American tragedy'. the Cutter incident and its implications for the Salk polio vaccine in New Zealand 1955-1960. Health History. 2009;11(2):42–61. 20481116

[41] Benison S. International medical cooperation: Dr. Albert Sabin, live poliovirus vaccine and the Soviets. Bull Hist Med. 1982; 56 (4): 460–83. 6760938

[42] Hampton L. Albert Sabin and the Coalition to Eliminate Polio from the Americas. Am J Public Health. 2009;99(1):34–44. 10.2105/AJPH.2007.117952 19008524 PMC2636601

[43] Porras MI, Báguena Cervellera MJ, Ballester Añón R. Spain and the international scientific conferences on polio, 1940s-1960s. Dynamis. 2010;30: 91–118. 10.4321/s0211-95362010000100004 20695166

[44] Ballester Añón R, Porras Gallo MI, Báguena Cervellera MJ. The Eradication of Poliomyelits in Spain: Projects, Obstacles, Achievements, Realities. Hygiea Internationalis. An Interdisciplinary Journal for the History of Public Health. 2015;11(1): 71–92.

[45] Ballester Añón R, Porras Gallo M.I. El significado histórico de las encuestas de seroprevalencia como tecnología de laboratorio aplicada a las campañas de inmunización. El caso de la poliomielitis en España. Asclepio. 2009;61(1): 55–80. 10.3989/asclepio.2009.v61.i1.272 19750612

[46] Tuells J. La batalla de Madrid por las vacunas antipoliomielitis (1963): ciencia, ideología y poder en la primera campaña de inmunización masiva en España. Gac Sanit. 2019; 33 (5): 480–4. 10.1016/j.gaceta.2018.05.005 10.1016/j.gaceta.2018.05.005 30031656

[47] Rodríguez Sánchez JA. Responsabilidades não asumidas: a poliomielite na Espahna (1954-1967) In: Nascimento DR, editor. A Historia da poliomielite. Rio de Janeiro: Garamons; 2010 p. 195–224.

[48] Jiménez Lucena I. El tifus exantemático de la posguerra española (1939-1943): el uso de una enfermedad colectiva en la legitimación del ‘Nuevo estado’. Dynamis. 1994;14: 131–145. 11624902

[49] Rodríguez Sánchez JA. Las secuelas sociales de la polio: los inicios del movimiento asociativo en España (1957-1975). Dynamis. 2012;32(2): 91–414. About this idea, we recommend watching the documentary Belis R, Armengou M. Polio, crónica de una negligencia. [Accessed 22 March 2019]. Available in: http://www.ccma.cat/tv3/alacarta/programa/Version-en-castellano-del-documental-Polio-cronica-de-una-negligencia/video/4900033/

